# Detection of tick-borne encephalitis virus in ear tissue and dried blood spots from naturally infected wild rodents

**DOI:** 10.1186/s13071-023-05717-0

**Published:** 2023-03-16

**Authors:** Emily L. Pascoe, Ankje de Vries, Helen J. Esser, Constantianus J. M. Koenraadt, Hein Sprong

**Affiliations:** 1 Laboratory of Entomology, Department of Plant Sciences, Wageningen University & Research, 6708 PB Wageningen, The Netherlands; 2 Laboratory for Zoonoses and Environmental Microbiology, National Institute for Public Health and Environment (RIVM), Antonie Van Leeuwenhoeklaan 9, P.O. Box 1, Bilthoven, The Netherlands; 3 Wildlife Ecology & Conservation Group, Wageningen University & Research, 6708 PB Wageningen, The Netherlands

**Keywords:** Tick-borne encephalitis virus, Reservoir host, Sampling, Surveillance

## Abstract

**Background:**

Tick-borne encephalitis virus (TBEV) can cause severe neurological disease in humans. Its geographical distribution is expanding in Western Europe with unresolved causes and spatial patterns, necessitating enhanced surveillance. Monitoring the virus in the environment is complicated, as it usually relies on destructive sampling of small rodents to test organs for TBEV, which in addition to ethical considerations also raises issues for long-term monitoring or longitudinal studies. Moreover, even when the virus is not detected in the blood or organs of the rodent, TBEV can still be transmitted from an infected tick to uninfected ticks feeding nearby. This is due to the ability of TBEV to replicate and migrate locally within the epidermis of small mammals, including those that do not appear to have systemic infection. This suggests that the virus may be detectable in skin biopsies, which has been confirmed in experimentally infected laboratory rodents, but it remains unknown if this sample type may be a viable alternative to destructively obtained samples in the monitoring of natural TBEV infection. Here we test ear tissue and dried blood spot (DBS) samples from rodents to determine whether TBEV-RNA can be detected in biological samples obtained non-destructively.

**Methods:**

Rodents were live-trapped and sampled at three woodland areas in The Netherlands where presence of TBEV has previously been recorded. Ear tissue (*n* = 79) and DBSs (*n* = 112) were collected from a total of 117 individuals and were tested for TBEV-RNA by real-time RT-PCR.

**Results:**

TBEV-RNA was detected in five rodents (4.3% of tested individuals), all of which had a TBEV-positive ear sample, while only two out of four of these individuals (for which a DBS was available) had a positive DBS. This equated to 6.3% of ear samples and 1.8% of DBSs testing positive for TBEV-RNA.

**Conclusions:**

We provide the first evidence to our knowledge that TBEV-RNA can be detected in samples obtained non-destructively from naturally infected wild rodents, providing a viable sampling alternative suitable for longitudinal surveillance of the virus.

**Graphical Abstract:**

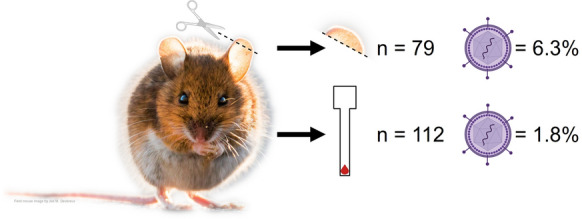

## Background

Tick-borne encephalitis virus (TBEV) is a Flavivirus that can cause severe and occasionally fatal neurological disease in humans [1, 2]. The distribution of TBEV is expanding and the number of human disease cases in Europe has been increasing over the last few decades [3], although recent vaccination programs have been very effective in limiting case numbers in some areas [5, 6]. Patterns of spread are unclear, but knowing where, when, and how the virus circulates in the environment is a crucial step in determining where interventions, e.g. vaccination programs, should be implemented. For TBEV this typically involves testing the organs of reservoir hosts (in Europe this is primarily small rodents such as *Apodemus* spp. and *Myodes* spp.) for viral RNA, but as this involves destructive sampling longitudinal studies are often not possible. Being able to test samples that can be obtained by non-destructive means would greatly expand the scope of such studies.

Testing for TBEV-binding antibodies is a non-destructive alternative to testing organs for RNA. Seroprevalence studies in deer and humans considered most at risk (e.g. forestry workers) have been effective in identifying new locations where TBEV is circulating [7, 8]. However, such studies provide limited information due to long-range movements of both humans and deer and cross-reactivity of antibodies induced by exposure (either natural or through vaccination) to other Flaviviruses [9]. Instead, some studies test for RNA in questing ticks; however, prevalences can be extremely low and are often < 1%, even at endemic “foci” where the virus is known to circulate [10–12]. Consequently, sampling rodents for TBEV-RNA can be preferable, as the percentage of samples in which the virus can be detected tends to be higher than for ticks and their home ranges are relatively small compared to other tick hosts such as deer [12]. However, it should be noted that home ranges of some small rodents can be multiple hectares in size and individuals have been known to disperse at least 2.5 km [13, 14]. Typically the brain, lungs, liver, or other organs are used for testing for virus presence in rodents [11, 15–18].

TBEV may also be detectable in samples that can be obtained non-destructively. The virus has been recorded in skin biopsies from experimentally infected laboratory rodents, even from tissue collected ~ 1 cm from the site of inoculation, because of the ability of the virus to replicate and migrate within the epidermis [19, 20]. Skin biopsies may even provide a more sensitive indication of TBEV presence as the virus can be detected in such samples even when it cannot be detected in the blood of an experimentally infected host [19, 20]. However, TBEV is yet to be successfully detected in ear tissue from naturally infected rodents. The virus has also been detected in whole blood collected from experimentally infected rodents [21] and in blood clots from rodents sampled as part of a surveillance study [11]. Here, we test ear tissue and dried blood spot samples from wild rodents to determine whether natural TBEV infections can be detected in biological samples obtained non-destructively. In other words, rodents could be released alive after sampling and followed up longitudinally via recapture if uniquely marked (e.g. with a Passive Integrated Transponder tag).

## Methods

### Sampling of rodents

Rodent sampling was performed in May–September 2021 at three woodland sites in The Netherlands where TBEV has previously been detected [12]. Heslinga live traps (Heslinga Traps, Groningen, The Netherlands) filled with hay for insulation and baited with grains, carrots and live mealworms were used to capture rodents. Rodent species, sex, and breeding status (juvenile, sub-adult, or adult; [22]) were recorded for all captured individuals. Briefly, individuals were recorded as juveniles if the post-juvenile moult had not yet occurred, individuals with adult pelage were considered adult if they were in breeding condition (descended testes for males and perforated vagina, pregnant, or nursing for females), or sub-adult if they did not meet these breeding condition criteria [22]. Rodents that weighed ≥ 20 g, were not evidently pregnant or nursing, and appeared to be in otherwise good health were eligible for biological sampling and were anaesthetised using isoflurane. Non-eligible rodents were released immediately at the site of capture. Once the rodent was anaesthetised, a small biopsy of ear tissue (~ 2 mm^2^) was cut from the tip of the pinna using surgical scissors and stored in 90% ethanol (as it was not originally intended for virus analyses). Blood was drawn by microhaematocrit capillary from the retro-orbital sinus and two or three drops were collected on Advantec® Nobuto Type I filter strips (Toyo Roshi Kaisha, Ltd., Japan). The Nobuto filter strip was stored in a dry microcentrifuge tube and is herein referred to as a dried blood spot (DBS). In some instances, blood was collected opportunistically, including from some individuals that were deemed ineligible for biological sampling, e.g. at the skin site where ticks were removed as part of another study. Rodents were allowed to recover from anaesthesia in a Makrolon™ cage filled with hay and grains and, once fully active and responsive, were released at the place of capture. All samples were stored at − 80 °C upon returning to the laboratory until further analysis. All handling procedures were approved by The Netherlands Ministry of Economic Affairs and by the Animal Experiments Committee of Wageningen University (2017.W-0049.013).

### Nucleic acid extraction and pathogen detection

Nucleic acid extraction and pathogen detection were performed in February 2022, approximately 6–9 months after samples were collected. Total nucleic acids (TNA) were extracted from ear tissue and DBSs using the MagNA Pure 96 DNA and Viral NA Small Volume Kit (Roche, Mannheim, Germany) in a MagNA Pure 96 Instrument (Roche). Ear tissue was first removed from the ethanol and dried. The ear tissue was then placed in 400 µl MagNA Pure 96 External Lysis Buffer together with 200 µl Minimum Essential Medium (MEM) (Gibco, Thermofisher Scientific, USA) in 2-ml tubes with Lysing Matrix Z (MP Biomedicals, Bio-Connect, The Netherlands). Samples were homogenised by beating for 40 s at 6.0 m/s (FastPrep-24™ 5G, MP Biomedicals), and the lysis buffer was transferred to a MagNA Pure 96 Processing Cartridge for TNA extraction. Nobuto filter strips with dried blood spots were incubated in 200 µl phosphate-buffered saline (PBS) and incubated for 20 min at 4 °C. The PBS was added to 275 µl lysis buffer with 18 mg/l yeast tRNA (Invitrogen, Thermofisher Scientific, USA) and vortexed before being transferred to a MagNA Pure 96 Processing Cartridge for TNA extraction. TBEV detection was performed by real-time RT-PCR [23]. This real-time RT-PCR amplifies a 68-bp fragment of the 3′ non-coding region. Samples with a ct score of < 40 and a characteristic amplification curve were considered positive.

## Results

We obtained 79 ear samples and 112 dried blood spots from a total of 117 rodents (Table 1). Both an ear sample and a DBS were collected from 74 of these individuals. Most ear samples were obtained from *Apodemus sylvaticus* (57%, *n* = 45), while 24% were from *Myodes glareolus* (*n* = 19) and 19% were from *Apodemus flavicollis* (*n* = 15). Similarly, more than half of the DBSs were taken from *A. sylvaticus* (54%, *n* = 61), 25% from *M. glareolus* (*n* = 28), 20% from *A. flavicollis* (*n* = 22), and one sample from an *Apodemus* that was not identified to species (Table 1).

**Table 1:** Overview of the number of samples collected and tested for tick-borne encephalitis virus from wild rodents at three locations in The Netherlands

Sample type collected	Rodent species	No. of rodents sampled		
		Location A	Location B	Location C
Ear and DBS	*Apodemus* sp. (unknown)	0	0	0
	*Apodemus flavicollis*	15	0	0
	*Apodemus sylvaticus*	10	19	13
	*Myodes glareolus*	9	1	7
	Total	34	20	20
Ear only	*Apodemus* sp. (unknown)	0	0	0
	*Apodemus flavicollis*	0	0	0
	*Apodemus sylvaticus*	0	2	1
	*Myodes glareolus*	1	0	1
	Total	1	2	2
DBS only	*Apodemus* sp. (unknown)	1	0	0
	*Apodemus flavicollis*	7	0	0
	*Apodemus sylvaticus*	4	6	9
	*Myodes glareolus*	4	2	5
	Total	16	8	14
Total number of samples per location		51	30	36

TBEV-RNA was detected at all three locations in a total of five rodents (4.3%), all of which had a TBEV-positive ear sample, while only two individuals had a positive DBS (DBSs were available for only four of these five TBEV-positive rodents) (Table 2). This equated to 6.3% of ear samples and 1.8% of DBSs testing positive for TBEV-RNA. Real-time RT-PCR ct scores ranged from 24.27–30.71 for ear samples and 25.38–32.94 for DBSs. All positive rodents were adults and comprised three *A. sylvaticus* (two males and one female) and two male *M. glareolus*. The TBEV-positive DBS came from a single *A. sylvaticus* (which had a ct score lower than that of the corresponding ear sample) and a *M. glareolus* (which had a ct score higher than that of the corresponding ear sample)*.* The percentage of TBEV-positive samples varied considerably by location; RNA was detected in 2.9–9.1% of tested ear samples and in 0–3.6% of DBSs.

**Table 2:** Overview of biological samples collected from rodents that tested positive by real-time RT-PCR for tick-borne encephalitis virus. Ct scores are provided in brackets

Species	Location	Sex	Breeding status	Ear tissue	Dried blood spot
*Apodemus sylvaticus*	B	Male	Adult	+ (30.70)	+ (25.38)
	C	Male	Adult	+ (30.71)	–
	B	Female	Adult	+ (29.12)	–
*Myodes glareolus*	C	Male	Adult	+ (25.73)	+ (32.94)
	A	Male	Adult	+ (24.27)	NA

## Discussion

We show for the first time to our knowledge that TBEV-RNA can be detected in ear tissue and dried blood spots from naturally infected wild rodents. Of 117 tested individuals, five were TBEV-positive, all of which had a positive ear sample and two of which also had a positive DBS. No rodents had a positive DBS but a negative ear sample. This supports earlier work which demonstrates that the virus can be present and replicate within skin tissue in experimentally infected laboratory rodents while being undetectable or almost undetectable in the blood [19, 20]. There is an increasing need to respond to the rise in human cases of tick-borne encephalitis and to the geographical expansion of TBEV throughout Europe [3]. An effective and appropriate response first requires an understanding of where and when the virus is circulating by monitoring known foci and identifying potential new ones [8]. Our results show that biological samples obtained non-destructively can be a viable alternative to testing rodent organs to monitor the presence of TBEV at natural foci, overcoming some issues associated with ethics and long-term monitoring.

TBEV has been detected in the skin of experimentally infected laboratory rodents, including in biopsies collected approximately 1 cm from the site of inoculation [17, 18]. These seminal studies determined that systemic infection of a reservoir host is not necessary for transmission of the virus to a feeding tick [20, 24, 25]. Instead, TBEV can be transmitted from infected to uninfected ticks feeding simultaneously nearby on a vertebrate host, even if that host is immune [17]. When the virus enters the vertebrate host at the bite site, viral particles are able to replicate and migrate locally using leukocytes within the epidermis to sites where other ticks are feeding, causing ticks that feed locally to become infected. This mechanism circumvents the need of systemic infection of the host [17, 18]. Vertebrate hosts have therefore been considered a “transient bridge” and “competent host” while ticks are regarded as both the vector and the true reservoir host of TBEV [4].

Currently, skin biopsies are seldom used in the monitoring of TBEV and previous studies have not detected TBEV in wild rodents from such samples, perhaps due to low prevalence of the virus in the sampled population [26]. However, in the present study we detected TBEV in 6.3% of tested ear samples. Our results are particularly promising when we consider that ear tissue samples were not primarily intended for RNA detection but rather to test for bacterial pathogens and therefore were not stored for optimal RNA preservation (i.e. they were stored in ethanol rather than RNAlater or by other methods more suitable for RNA preservation). It is not possible to understand if or by how much these suboptimal storage conditions impacted on RNA preservation and subsequent detection. However, as TBEV-RNA was still detectable in some samples it is possible that because they were preserved almost immediately at − 80 °C and tested for TBEV within ~ 9 months of collection, any adverse effects of ethanol storage were somewhat limited. While we did not compare the sensitivity of ear or DBS samples to that of organs which are typically tested for the virus or to samples from individuals with a known infection status, we still believe that ear tissue in particular could provide an alternative to destructive sampling. Notably, in another study conducted within The Netherlands in which organs from 320 rodents were tested, including 53 rodents collected at two of the same sites sampled here, TBEV-RNA was detected in a considerably lower percentage of individuals (0.9%, *n* = 3) [12]. We recognise, however, that local prevalence of TBEV within the rodent population can vary considerably according to TBEV subtype, year, location, and rodent species, with prevalences of > 60% recorded in rodents in some studies [11, 13–16].

Multiple studies have successfully used blood dried on filter paper to detect antibodies for tick-borne pathogens, including TBEV and other viruses [27–30]. Pathogens (e.g. hantavirus and TBEV-RNA) have also been detected in organ/tissue samples preserved on dry filter paper [31]. Moreover, prior to our study there was compelling evidence to suggest that TBEV-RNA could be successfully detected in dried blood spots. The virus had been found in whole blood of rodents stored by other methods (e.g. [11, 19]) and RNA from TBEV cell culture supernatant was easily recovered from dried filter paper. However, we provide the first example of using small volumes of whole blood dried on filter paper to detect TBEV in naturally infected wild-captured rodents. This is a particularly convenient method of collecting and storing blood samples under field conditions, when, for example, prompt freezing or centrifugation of samples to obtain a blood clot is not possible. Despite this, the method appeared inferior to testing ear tissue for TBEV presence, as TBEV was not detected in all DBS samples from rodents that had a positive ear sample, although ct scores for positive samples were comparable for ear and DBS samples. This also supports research conducted on laboratory animals which demonstrates that active experimental infection of TBEV can occur without resulting in a detectable systemic infection of the host [17, 18], although we acknowledge that more controlled work would be necessary to further confirm this in wild rodents.

## Conclusions

Our study provides evidence for the first time that TBEV-RNA can successfully be detected in samples obtained non-destructively from naturally infected wild rodents, providing a viable sampling alternative suitable for longitudinal or long-term surveillance of the virus.

## Data Availability

All data generated or analysed during this study are included in this published manuscript.
